# Predictors of 30-Day Readmission in Patients With Cirrhosis: A Retrospective Cohort Study

**DOI:** 10.7759/cureus.104868

**Published:** 2026-03-08

**Authors:** Noor Albusta, Ali Yusuf, Hussain Alrahma

**Affiliations:** 1 Internal Medicine, Beth Israel Lahey Health, Cambridge, USA; 2 General Surgery, Salmaniya Medical Complex, Manama, BHR; 3 Medicine, Bahrain Government Hospitals, Manama, BHR; 4 Gastroenterology and Hepatology, Bahrain Government Hospitals, Manama, BHR

**Keywords:** ascites, cirrhosis, hepatic encephalopathy, meld scores, readmission

## Abstract

Introduction: Cirrhosis is associated with significant morbidity and frequent hospital readmissions due to recurrent complications. Thirty-day readmission is an important quality metric linked to increased healthcare costs and mortality. Identifying predictors of early readmission may help guide targeted interventions.

Methods: We conducted a retrospective cohort study of adult patients (≥18 years) with cirrhosis admitted to a tertiary care center in Bahrain between January 1, 2023, and December 31, 2025. Demographic, clinical, and laboratory data were collected from electronic medical records. The primary outcome was 30-day hospital readmission. Multivariate logistic regression was used to identify independent predictors.

Results: A total of 527 patients were included, with a mean age of 56.8 ± 13.2 years; 324 (61.5%) were male. The most common etiologies were metabolic dysfunction-associated steatotic liver disease (203 (38.5%)), alcohol-related liver disease (143 (27.1%)), and viral hepatitis (130 (24.7%)). Ascites was present in 329 patients (62.4%) and hepatic encephalopathy in 184 patients (34.9%). Hyponatremia (serum sodium < 130 mmol/L) occurred in 148 patients (28.1%), and prior hospitalization within six months occurred in 211 patients (40.0%). The mean Model for End-Stage Liver Disease (MELD) score was 16.7 ± 6.3. The 30-day readmission rate was 144 (27.3%). Independent predictors of readmission included a higher MELD score (odds ratio (OR) 1.08 per point increase, 95% confidence interval (CI) 1.04-1.12, p < 0.001), ascites (OR 2.14, 95% CI 1.38-3.31, p = 0.001), hepatic encephalopathy (OR 1.89, 95% CI 1.21-2.95, p = 0.005), hyponatremia (OR 1.76, 95% CI 1.12-2.77, p = 0.014), and prior hospitalization (OR 2.58, 95% CI 1.65-4.04, p < 0.001).

Conclusion: Thirty-day readmission among patients with cirrhosis is common and is primarily driven by disease severity and complications. Higher MELD score, ascites, hepatic encephalopathy, hyponatremia, and prior hospitalization were independently associated with increased readmission risk. Identifying high-risk patients may allow implementation of targeted interventions, including optimization of medical management and early outpatient follow-up, to reduce readmission and improve outcomes.

## Introduction

Cirrhosis represents the final stage of chronic liver disease and is associated with significant morbidity, mortality, and healthcare utilization [1,2]. The global burden of cirrhosis continues to increase, with patients frequently developing complications such as ascites, hepatic encephalopathy, variceal bleeding, and infections, often resulting in repeated hospitalizations [1,3]. The development of ascites, commonly the first decompensation-defining event, is associated with a reduction in five-year survival from 80% to 30% [4].

Hospital readmission, particularly within 30 days of discharge, has emerged as an important quality metric and is associated with increased healthcare costs and adverse clinical outcomes [3,4]. Reported 30-day readmission rates in cirrhosis range from 20% to 37%, reflecting the progressive nature of the disease [5]. A large nationwide study of 123,011 patients with decompensated cirrhosis found a 30-day readmission rate of 27%, with 79.6% of patients readmitted with liver-related diagnoses [1]. Another multistate population-based cohort study reported 30-day and 90-day readmission rates of 12.9% and 21.2% overall, with rates increasing to 24.2% and 35.9%, respectively, among patients with more than three complications of cirrhosis [5].

The economic burden of cirrhosis-related readmissions is substantial. A nationwide analysis estimated the weighted cumulative national cost of index admissions at $1.8 billion, compared to $0.5 billion for readmissions, highlighting the significant financial impact on healthcare systems [2]. Furthermore, readmissions are associated with increased mortality risk, with studies demonstrating that patients with early readmission have significantly higher mortality rates compared to those not readmitted [6].

Identifying predictors of early readmission is essential to improve patient outcomes and optimize healthcare resource utilization [3,4]. Prior studies have identified disease severity, as measured by the Model for End-Stage Liver Disease (MELD) score, and complications of cirrhosis as major contributors to readmission risk [1,5]. The MELD score, which incorporates serum creatinine, bilirubin, and international normalized ratio (INR), has been consistently associated with increased readmission risk across multiple studies [1,3,7]. Specific complications including hepatic encephalopathy, ascites, variceal bleeding, and spontaneous bacterial peritonitis have also been identified as significant predictors [4,8].

Despite the growing body of literature on cirrhosis readmissions, data from the Middle East, particularly Bahrain, remain limited. Understanding regional patterns and predictors of readmission is important given potential differences in patient demographics, etiology of liver disease, healthcare delivery systems, and access to specialized care. The primary objective of this study was to evaluate predictors of 30-day readmission among patients with cirrhosis admitted to a tertiary care center in Bahrain. The secondary objective was to identify high-risk patients who may benefit from targeted interventions aimed at reducing readmission.

## Materials and methods

Study design and setting

This retrospective cohort study was conducted at a tertiary care center in Bahrain (Bahrain Government Hospitals), including patients admitted between January 1, 2023, and December 31, 2025. Ethical approval was obtained from the institutional review board at Bahrain Government Hospitals, and the requirement for informed consent was waived due to the retrospective nature of the study.

Study population

Inclusion Criteria

Inclusion criteria were adults aged ≥18 years; a diagnosis of cirrhosis based on clinical, radiological, or histological findings; and an index hospital admission during the study period.

Exclusion Criteria

Exclusion criteria included liver transplant recipients, patients with advanced hepatocellular carcinoma beyond transplant criteria, patients who died during the index admission, and those with incomplete medical records.

Data collection

Data were extracted from electronic medical records and included demographic, clinical, laboratory, and hospitalization-related variables. Demographic variables included age, sex, and etiology of cirrhosis, classified as alcohol-related liver disease, metabolic dysfunction-associated steatotic liver disease (MASLD), viral hepatitis, or other causes. Clinical variables included the presence of ascites, hepatic encephalopathy, variceal bleeding, and spontaneous bacterial peritonitis. Laboratory parameters included serum sodium, creatinine, total bilirubin, INR, and serum albumin. Laboratory data were used to calculate the MELD score at the time of admission. The MELD score was originally described by Malinchoc et al. [9]. Additional variables included prior hospitalization within six months.

Outcome measure

The primary outcome was 30-day hospital readmission, defined as any unplanned admission within 30 days of discharge from the index hospitalization. Readmissions were identified through review of electronic medical records and hospital administrative databases.

Statistical analysis

Continuous variables were expressed as mean ± standard deviation or median (interquartile range), as appropriate based on data distribution, while categorical variables were presented as frequencies and percentages. Comparisons between the readmitted and non-readmitted groups were performed using Student’s t-test or the Mann-Whitney U test for continuous variables, and the chi-squared test or Fisher’s exact test for categorical variables, as appropriate. Variables with a p-value < 0.10 in univariate analysis were included in a multivariate logistic regression model to identify independent predictors of 30-day readmission. Results were reported as odds ratios (OR) with 95% confidence intervals (CI). A p-value < 0.05 was considered statistically significant. All statistical analyses were performed using SPSS software (version 28; IBM Corp., Armonk, NY, USA). Missing data were assessed during data extraction. Variables with minimal missing values were analyzed using complete-case analysis, and no variable included in the final multivariate model had substantial missing data.

## Results

Baseline characteristics

**Figure 1:**
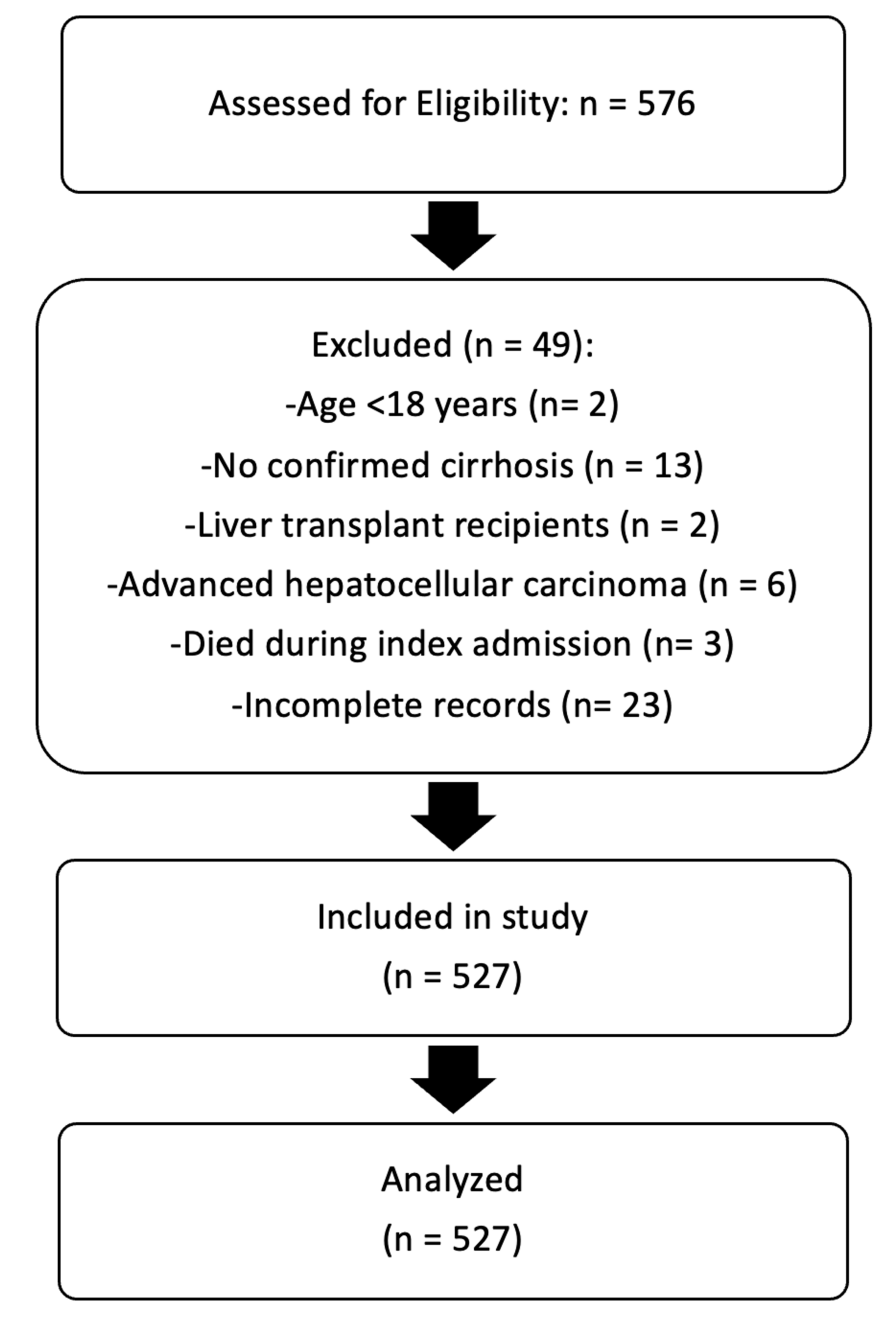
Cohort flowchart illustrating inclusion and exclusion criteria and final sample size for analysis

A total of 527 patients were included in the analysis (Figure 1).

The mean age was 56.8 ± 13.2 years, and 324 patients (61.5%) were male. The most common etiologies of cirrhosis were MASLD in 203 patients (38.5%), alcohol-related liver disease in 143 patients (27.1%), viral hepatitis in 130 patients (24.7%), and other causes in 51 patients (9.7%). Hyponatremia (serum sodium < 130 mmol/L) was present in 148 patients (28.1%), and 211 patients (40.0%) had a prior hospitalization within six months. Features of hepatic decompensation included ascites in 329 patients (62.4%), hepatic encephalopathy in 184 patients (34.9%), variceal bleeding in 114 patients (21.6%), and spontaneous bacterial peritonitis in 83 patients (15.8%). Patients may have more than one hepatic decompensation event; therefore, percentages are not mutually exclusive, and totals may exceed 100%. The mean MELD score was 16.7 ± 6.3 (Table 1).

**Table 1:** Baseline characteristics of the study population SD: standard deviation; MELD: Model for End-Stage Liver Disease

Characteristic	Value
Age, mean ± SD (years)	56.8 ± 13.2
Male sex, n (%)	324 (61.5%)
Etiology of cirrhosis	
Metabolic dysfunction-associated steatotic liver disease, n (%)	203 (38.5%)
Alcohol-related liver disease, n (%)	143 (27.1%)
Viral hepatitis, n (%)	130 (24.7%)
Other causes, n (%)	51 (9.7%)
Hepatic decompensation	
Ascites, n (%)	329 (62.4%)
Hepatic encephalopathy, n (%)	184 (34.9%)
Variceal bleeding, n (%)	114 (21.6%)
Spontaneous bacterial peritonitis, n (%)	83 (15.8%)
MELD score, mean ± SD	16.7 ± 6.3

Readmission rate

The overall 30-day readmission rate was 27.3% (n = 144). Patients who were readmitted within 30 days differed significantly from those who were not readmitted across several baseline characteristics. Readmitted patients had higher mean MELD scores (18.9 ± 6.1 vs. 15.7 ± 6.1, p < 0.001), higher prevalence of ascites (113 (78.5%) vs. 216 (56.4%), p = 0.002), hepatic encephalopathy (75 (52.1%) vs. 109 (28.5%), p = 0.004), and hyponatremia (serum sodium < 130 mmol/L) (59 (41.0%) vs. 89 (23.2%), p = 0.01). Readmitted patients were also more likely to have had a prior hospitalization within six months (84 (58.3%) vs. 127 (33.2%), p < 0.001), lower mean serum albumin (2.8 ± 0.6 vs. 3.2 ± 0.7 g/dL, p = 0.03), and higher mean serum creatinine (1.4 ± 0.6 vs. 1.1 ± 0.5 mg/dL, p = 0.02). The two groups did not differ significantly with respect to age (57.6 ± 12.8 vs. 56.4 ± 13.4 years, p = 0.42) or sex (male: 94 (65.3%) vs. 230 (60.1%), p = 0.31) (Table 2).

**Table 2:** Characteristics stratified by 30-day readmission status SD: standard deviation; MELD: Model for End-Stage Liver Disease

Variable	Readmitted (n = 144)	Not readmitted (n = 383)	p-value
Age, years (mean ± SD)	57.6 ± 12.8	56.4 ± 13.4	0.42
Male sex, n (%)	94 (65.3%)	230 (60.1%)	0.31
MELD score (mean ± SD)	18.9 ± 6.1	15.7 ± 6.1	<0.001
Ascites, n (%)	113 (78.5%)	216 (56.4%)	0.002
Hepatic encephalopathy, n (%)	75 (52.1%)	109 (28.5%)	0.004
Hyponatremia (<130 mmol/L), n (%)	59 (41.0%)	89 (23.2%)	0.01
Prior hospitalization (6 months), n (%)	84 (58.3%)	127 (33.2%)	<0.001
Serum albumin, g/dL (mean ± SD)	2.8 ± 0.6	3.2 ± 0.7	0.03
Serum creatinine, mg/dL (mean ± SD)	1.4 ± 0.6	1.1 ± 0.5	0.02

Univariate analysis

Univariate analysis demonstrated that higher MELD score (p < 0.001), presence of ascites (p = 0.002), hepatic encephalopathy (p = 0.004), hyponatremia (serum sodium < 130 mmol/L) (p = 0.01), prior hospitalization within six months (p < 0.001), lower serum albumin (p = 0.03), and higher serum creatinine (p = 0.02) were significantly associated with 30-day readmission. Variables with a p-value < 0.10 were subsequently included in the multivariate logistic regression model to identify independent predictors of readmission.

Multivariate analysis

Independent predictors of 30-day readmission identified through multivariate logistic regression analysis are summarized in Table 3.

**Table 3:** Multivariate logistic regression analysis of predictors of 30-day readmission in patients with cirrhosis MELD: Model for End-Stage Liver Disease

Variable	Odds ratio (OR)	95% confidence interval	p-value
MELD score (per point)	1.08	1.04-1.12	<0.001
Ascites	2.14	1.38-3.31	0.001
Hepatic encephalopathy	1.89	1.21-2.95	0.005
Sodium < 130 mmol/L	1.76	1.12-2.77	0.014
Prior hospitalization (6 months)	2.58	1.65-4.04	<0.001

## Discussion

In this retrospective cohort study of 527 patients with cirrhosis admitted to a tertiary care center in Bahrain, the 30-day readmission rate was 27.3%, consistent with previously reported rates ranging from 20% to 37% in the literature [5,10,11]. Disease severity, as reflected by the MELD score, and complications of cirrhosis, including ascites, hepatic encephalopathy, and hyponatremia, were identified as significant independent predictors of readmission. Additionally, prior hospitalization within six months was strongly associated with increased readmission risk. These findings align with prior studies across diverse populations and healthcare systems, suggesting that the primary drivers of readmission in cirrhosis are related to disease progression and complications rather than regional factors [12,13].

A higher MELD score was independently associated with increased readmission risk (OR 1.08 per point increase), reflecting the impact of advanced liver dysfunction on clinical outcomes [14]. This finding is consistent with multiple prior studies demonstrating that the MELD score is a robust predictor of readmission across diverse patient populations [8,15]. A large multicenter North American study found that patients with worse MELD scores were more likely to be readmitted within three months, with the MELD score being a consistent predictor across all readmission types [8]. The MELD score integrates key markers of hepatic synthetic function (INR, bilirubin) and renal function (creatinine), providing a comprehensive assessment of disease severity that correlates with mortality and morbidity risk [15].

The presence of ascites was a strong independent predictor of readmission (OR 2.14), likely due to its recurrent nature and need for ongoing management [1,4,5]. Ascites is commonly the first decompensation-defining event in cirrhosis and is associated with a reduction in five-year survival from 80% to 30% [5]. Patients with ascites are prone to additional complications including spontaneous bacterial peritonitis, hepatorenal syndrome, and electrolyte abnormalities, contributing to clinical decline and readmission risk [5,16]. A recent study found that preventable readmissions in cirrhosis were often related to paracentesis timeliness and diuretic adjustment monitoring, highlighting opportunities for intervention [2]. Optimal management of ascites requires careful titration of diuretics, sodium restriction, and timely large-volume paracentesis when indicated, along with close outpatient monitoring to prevent decompensation [5,16].

Hepatic encephalopathy was independently associated with readmission (OR 1.89), consistent with its recognition as one of the strongest predictors of readmission in cirrhosis [5,8,16]. A multistate population-based study found that hepatic encephalopathy was most strongly associated with readmission within 30 and 90 days (OR 1.77) [5]. The recurrent nature of hepatic encephalopathy, combined with its impact on medication adherence, fall risk, and quality of life, contributes to the high readmission rate in affected patients [15,16]. Quality improvement initiatives incorporating goal-directed lactulose therapy and rifaximin for overt hepatic encephalopathy have demonstrated significant reductions in 30-day readmissions [17]. A prospective study showed that during an electronic phase incorporating these interventions, the proportion of patients with grade >2 hepatic encephalopathy and 30-day readmission decreased from 48.9% to 26.0% (p = 0.0003) [17].

Hyponatremia (serum sodium < 130 mmol/L) was independently associated with readmission (OR 1.76), consistent with its role as a marker of circulatory dysfunction and advanced portal hypertension [12,13]. Hyponatremia reflects worsening hemodynamic status as cirrhosis advances, with patients having serum sodium ≤ 130 mEq/L at increased risk for developing hepatic encephalopathy (OR 3.4), hepatorenal syndrome (OR 3.5), and spontaneous bacterial peritonitis (OR 2.4) [12]. The prognostic significance of hyponatremia is well established, with studies demonstrating increased in-hospital and waitlist mortality in hyponatremic patients [13,14]. Management of hyponatremia in cirrhosis is challenging and typically involves fluid restriction, discontinuation or dose reduction of diuretics, and, in select cases, albumin infusion or vasopressin receptor antagonists [13,16].

Patients with prior hospitalizations within six months had significantly higher odds of readmission (OR 2.58), suggesting a subgroup with unstable disease and high healthcare utilization [2,8]. This finding is consistent with prior studies identifying recent hospitalization as a strong predictor of subsequent readmission [1,2]. Patients with frequent hospitalizations may have more advanced disease, poor social support, medication non-adherence, or inadequate outpatient follow-up, all of which contribute to readmission risk [2,3]. A prospective study found that preventable readmission was independently associated with admission in the prior 30 days (OR 3.45) [2].

These findings highlight the importance of targeted interventions focused on high-risk patients to reduce readmission rates and improve outcomes in cirrhosis. Multidisciplinary care coordination, including specialized teams of nurses and hepatologists, has been shown to reduce 30-day and 12-month readmission rates, decrease mortality, and lower healthcare costs by facilitating transitions of care, optimizing medication management, and ensuring timely follow-up [10,16]. Optimization of hepatic encephalopathy management, particularly through goal-directed lactulose therapy and the use of rifaximin, has been associated with significantly lower odds of readmission, likely through prevention of recurrence and improved adherence to therapy [16,17]. Effective management of ascites, including appropriate diuretic adjustment, patient education, and timely access to paracentesis, is also critical, as preventable readmissions are frequently related to suboptimal ascites management [2,5,16]. Early outpatient follow-up, ideally within 7-14 days after discharge, allows for prompt reassessment, medication review, and early intervention, thereby reducing the risk of clinical deterioration and rehospitalization [16]. In addition, health information technology interventions, including digital tools focused on medication adherence and symptom monitoring, have demonstrated reductions in avoidable readmissions in randomized controlled trials [18]. Integration of evidence-based cirrhosis care pathways into electronic medical records further supports standardized management and has been associated with improved outcomes, including reduced mortality and higher rates of appropriate interventions [9]. Collectively, these strategies emphasize the importance of structured, proactive care models in mitigating the burden of readmissions in patients with cirrhosis [9,16,18].

While this study identified multiple predictors of readmission, it is important to recognize that not all readmissions are preventable. A recent prospective study with systematic adjudication found that only 12% of 30-day readmissions in cirrhosis were deemed preventable, with most readmissions being related to disease progression and unavoidable complications [2]. However, preventable readmissions were often related to ascites and hepatic encephalopathy management, suggesting that targeted interventions in these areas may have the greatest impact [2,17]. The challenge lies in identifying which patients are at the highest risk for preventable readmissions and implementing appropriate interventions.

Limitations

This study has several limitations that should be acknowledged. First, its retrospective design may be subject to missing data, selection bias, and unmeasured confounding. Important factors such as medication adherence, social support, health literacy, and socioeconomic status were not systematically assessed but may significantly influence readmission risk. Second, the study was conducted at a single tertiary care center in Bahrain, which may limit generalizability to other settings, particularly community hospitals or healthcare systems with different resources and patient populations. Third, readmissions to other hospitals outside the study center may not have been captured, potentially underestimating the true readmission rate. Fourth, the study did not assess the preventability of readmissions or distinguish between liver-related and non-liver-related readmissions, which may have different predictors and prevention strategies. Fifth, specific interventions such as rifaximin use, lactulose dosing, and discharge planning quality were not systematically evaluated, limiting the ability to identify modifiable factors. Finally, longer-term outcomes beyond 30 days, including 90-day readmissions and mortality, were not examined.

## Conclusions

Thirty-day readmission among patients with cirrhosis remains a significant clinical challenge and reflects the complex, dynamic nature of advanced liver disease. Readmissions are often driven by disease progression, complications, and gaps in transitional care. Identifying patients at higher risk provides an opportunity to implement targeted strategies, including optimized inpatient management, comprehensive discharge planning, and structured outpatient follow-up. Strengthening care coordination and early post-discharge support may help reduce preventable readmissions and improve overall patient outcomes.
